# Ultrasound or Sectional Imaging Techniques as Screening Tools for Hepatocellular Carcinoma: Fall Forward or Move Forward?

**DOI:** 10.3390/jcm10050903

**Published:** 2021-02-25

**Authors:** Zeno Sparchez, Rares Craciun, Cosmin Caraiani, Adelina Horhat, Iuliana Nenu, Bogdan Procopet, Mihaela Sparchez, Horia Stefanescu, Tudor Mocan

**Affiliations:** 1 3rd Medical Department, “Iuliu Hatieganu” University of Medicine and Pharmacy, 400162 Cluj-Napoca, Romania; zsparchez@elearn.umfcluj.ro (Z.S.); horhat.adelina@elearn.umfcluj.ro (A.H.); nenu.iuliana@elearn.umfcluj.ro (I.N.); bogdanprocopet@elearn.umfcluj.ro (B.P.); mocan.tudor@umfcluj.ro (T.M.); 2 Regional Institute of Gastroenterology and Hepatology, 400162 Cluj-Napoca, Romania; horia.stefanescu@irgh.ro; 3 Department of Medical Imaging, “Iuliu Hatieganu” University of Medicine and Pharmacy, 400012 Cluj-Napoca, Romania; cosmin.caraiani@umfcluj.ro; 4 Paediatric Department, “Iuliu Hatieganu” University of Medicine and Pharmacy, 400012 Cluj-Napoca, Romania; spirchez.mihaela@umfcluj.ro

**Keywords:** hepatocellular carcinoma, ultrasound surveillance, sectional imaging, early detection

## Abstract

Hepatocellular carcinoma (HCC) is probably the epitome of a screening target, with a well-defined high-risk population, accessible screening methods, and multiple curative-intent treatments available for early disease. Per major societies guideline consensus, biannual ultrasound (US) surveillance of the at-risk patients is the current standard of care worldwide. Yet, despite its documented success in the past decades, this standard is far from perfect. While the whole community is working to further tighten the knots, a worrying number of cases still slip through this safety net. Consequently, these patients lose their chance to a curative solution which leads to a high disease burden with disproportionate mortality. While US will probably remain the fundamental staple in the screening strategy, key questions are seeking better answers. How can its caveats be addressed, and the technique be improved? When are further steps needed? How to increase accuracy without giving up on accessibility? This narrative review discusses the place of US surveillance in the bigger HCC picture, trying to navigate through its strengths and limits based on the most recent available evidence.

## 1. Topic Overview—Why Do We Need HCC Surveillance?

As of 2020, hepatocellular carcinoma (HCC) represents a major cause of morbidity and mortality, especially among patients with chronic liver disease. Available reports rank primary liver cancer as the sixth most common type of malignancy, disproportionally accounting for the fourth place in cancer-related mortality [1]. While these figures appear to be relatively stable throughout the recent years, the field is facing unprecedented effervescence, with rapid shifts occurring on multiple levels of knowledge.

Thus, the most consequential clinical dilemmas remain: why is HCC surveillance needed and what is the best approach to do it? The reason for them being the most consequential is straightforward. Even though new data is constantly emerging regarding new therapeutic regimens, the beneficial increments are still relatively small. HCC remains a diagnosis marked by high fatality rates, as proven by an incidence to mortality ratio desolately close to 1 [2]. In this light, the cornerstone of HCC survival remains early detection. This statement is backed by clear-cut data, early diagnosis rendering a 5-year survival exceeding 70%, compared to intermediate and advanced stage diagnosis which leads to a dismal, less than 20%, survival [3,4]. More explicitly, new data has shown that patients diagnosed and treated in the earliest Barcelona Clinic Liver Cancer (BCLC)—0 stage had an 86.2% 5-year survival, with a significant decrease in survival with upstaging −69.0% for BCLC A and 49.9% for BCLC B [5]. These figures dramatically drop when analyzing survival for late stage, BCLC C and D HCC, where survival is rarely above 12 months and 3 months, respectively [3,6].

Cancer surveillance programs aim to detect tumors at an early stage, when they are treatable with curative intent, thus improving survival [7]. However, the evidence for a survival benefit associated with HCC screening in patients with cirrhosis remains controversial due to the paucity of level I evidence to prove it [8]. There are only two randomized controlled trials, dichotomizing patients into screening and no screening groups, published on a large Chinese Hepatitis B Virus (HBV) cirrhosis cohort, one of them showing a 37% decrease in liver cancer-related mortality for the screening group [9].

Most of the research investigating HCC surveillance and mortality consists of observational cohort studies, the majority being retrospective. A meta-analysis of 47 observational studies found that surveillance improved detection of early-stage HCC (odds ratio [OR]-2.08), increased curative treatment rates (OR-2.24), and improved survival (OR-1.90), but there are several potential caveats [10]. In this light, the strength of the evidence supporting these screening programs remains disputable, especially with regards to mortality [11].

Future randomized controlled trials (RCTs) would provide the finest evaluation of surveillance impact, but appear to be unethical by all current standards, as most patients prefer surveillance [12]. Though high-quality data are lacking, there are currently no proposed alternatives to surveillance. With important improvements in HCC treatment over recent years, surveillance is likely to be beneficial.

The current standard of practice for HCC surveillance is bi-annual ultrasound (US) screening, per major society guidelines consensus [13,14,15,16,17]. However, the effectiveness of the ongoing screening strategies can be significantly improved.

The main advantages of US surveillance are its accessibility, non-invasive character, repeatability, and patient tolerance. Yet, even if its effectiveness is assumed based on empirical grounds, the enrollment in regular follow-up programs remains astoundingly low, even in developed countries with otherwise praised medical systems. Available reports suggest that less than one-third of the patients with cirrhosis are either included in or compliant with HCC screening programs [18,19], with further discrepancies occurring with regards to social status or liver disease etiology [20,21]. Not least, data suggest that less than half of patients with cirrhosis are regularly followed-up in specialized hepatology units, which places an increased burden on primary care providers to stay knowledgeable and updated with the diagnostic and therapeutic approach to an already complex issue [22].

## 2. Surveillance Techniques and Ongoing Strategies for HCC

Surveillance of HCC requires repeated applications of screening tools in patients at risk, aiming to reduce disease-related mortality. The outcome of surveillance is determined by the incidence of HCC in the target population, the availability and acceptance of efficient diagnostic tests, and the effective treatment [23]. The techniques used in HCC surveillance include imaging and serological examinations. The most widely used imaging method is abdominal ultrasound (US). It is indicated in patients at risk of developing HCC, notably cirrhotic patients and patients with chronic HBV infection, as long as their liver function is sufficient to allow for a therapeutic approach [24,25].

Currently, US surveillance is recommended by the European Association for the Study of the Liver (EASL), the American Association for the Study of Liver Diseases (AASLD), and the Asian Pacific Association for the Study of the Liver (APASL) [13,14,15,16,17]. Multidetector CT (Computed Tomography) or dynamic MR (Magnetic Resonance) imaging are generally not cost-effective for surveillance but may be used in specific circumstances. Their indications and limitations will be discussed later in our review.

The use of tumor markers (especially alpha-fetoprotein, AFP) alone is currently not recommended for HCC screening, but the combination of AFP and B-mode US is endorsed by Eastern countries [15,16]. To increase accuracy, a value > 200 ng/dl is recommended as a threshold for surveillance purposes [16]. However, in patients with previous curative treatment for HCC, or those successfully treated with antivirals, the cut-off of 20 ng/dl appears to be more valuable [26]. Interesting new data shows that longitudinal changes in AFP may have better accuracy than a single value > 20 ng/dl [27].

The combination of US + AFP can lead to a 6% gain in the early HCC detection rate, but at the cost of false-positive results [28]. A large meta-analysis showed no benefit in early detection and receipt of curative therapy rates if AFP was added to B-mode US surveillance [10]. In contrast, in a population exceeding 1500 cirrhotic patients, AFP > 20 ng/dl used together with US surveillance increased the sensitivity of HCC detection up to 99.2% [29].

Other serological tests that have been used or are under investigation for HCC diagnosis are lens culinaris agglutinin-reactive fraction of AFP (AFP-L3) and des-gamma-carboxyprothrombin (DCP) [14]. A Korean study revealed that, when combined with AFP, AFP-L3 significantly increased the detection sensitivity from 62% (AFP alone) to 79% (AFP and AFP-L3) at a very early stage. The Japanese Society of Hepatology uses AFP in combination with DCP as a surveillance technique. DCP seems to be correlated with tumor size, with superior performance to AFP, and is also associated with a more aggressive phenotype [15].

Several other biomarkers have been proposed as a screening tool in HCC including proteins (e.g., mRNAs), metabolites, extracellular vesicles, circulating free DNA, or circulating tumor cells [30]. Discussing all these biomarkers is beyond the purpose of this paper. Nevertheless, from bench to bedside there is still a long road ahead.

With regards to optimal surveillance schedule, most of the available data converges towards a 6-month interval. The previously mentioned Italian database revealed a significant decrease in failure rates from annual to bi-annual visits (41.3% vs. 32.2%), regardless of other features [31]. These findings are reinforced by a large-scale retrospective analysis from Taiwan [32], which compared bi-annual follow-up, to annual and less frequent visits and concluded that shorter visit intervals were associated with lower 5-year mortality. However, the benefit of decreasing the interval below 6-months is questionable, as data suggest that HCC detection (<3 cm) and overall survival did not significantly improve if a 3-month interval was implemented [33]. No difference in either HCC incidence or in prevalence of tumors ˂ 30 mm in diameter (79% versus 70%, *p* ˂ 0.30) was observed between the randomized groups [33]. The 6-month interval is therefore currently recommended by all major society guidelines, as previously mentioned.

Finally, it is important whom to offer the surveillance program for HCC. The at-risk population has been well-defined and comprises: all cirrhotic patients, regardless of etiology and disease severity (except for Child–Pugh C patients—only those awaiting liver transplantation), non-cirrhotic HBV patients at intermediate or high risk of HCC and non-cirrhotic F3 patients, regardless of etiology [14]. Risk among those populations is very variable and can be further stratified and refined using information gained through liver stiffness and risk scores assessment (see below).

## 3. Ultrasound Aspects of HCC Discovered during Screening

We can all agree that US is a powerful screening tool for HCC. It is a noninvasive and literally risk-free procedure; inexpensive and ubiquitously available; and not least, it is a patient-friendly procedure [34]. However, several clinical dilemmas still exist even now, after several decades of US screening in HCC.

What are the ultrasound features of HCC? What should one be looking at? The aim of US examination in the screening process is to detect nodules that may represent early or very early HCC. When searching for nodules, two main features are important: the US aspect and the size of the nodule. Most of the small HCCs (<2 cm) are hypoechoic, but HCC may also appear as an iso or even as a hyperechoic nodule. One study that included 153 consecutive small HCC patients found that 76.4% were hypoechoic, 17% were hyperechoic, 3.3% were isoechoic and 3.3% had nodule-in nodule pattern. This echogenicity distribution was similar in the 2–3 cm range. Patients with a hyperechoic pattern displayed a trend towards lower AFP levels, younger age, and a higher prevalence of hepatitis C—related cirrhosis. The prevalence of well-differentiated tumors was identical (55.6% and 54.5%) in the hypoechoic and hyperechoic subgroups [35]. Another study has shown that the prevalence of hyperechoic small HCC nodules may be as high as 24% [36]. The main differential diagnosis includes haemangioma and dysplastic nodules. Considering this, small hyperechoic lesions detected in cirrhotic livers should be managed similarly to hypoechoic nodules [35].

In clinical practice we can encounter other US features such as: (a) nodules with a halo; these nodules tend to have a higher chance of becoming HCC; (b) if one nodule has ill-defined margins and during follow-up transforms into a nodule with well-defined margins the probability of HCC increases; (c) the appearance of vasculature on color flow US during follow-up is also a worrisome feature; and (d) hyperechoic nodules have a lower chance of becoming HCC [37]. Other US features of HCC discovered during surveillance are large, multinodular, diffuse tumors with or without portal vein thrombosis (PVT). Sometimes the only US sign of an HCC is PVT [38].

In a multicenter study the size of nodules detected during an active surveillance was mostly either <2 cm (42,7%) or between 2–3 cm (40.3%), only 17% being larger than 3 cm (39). The probability for one nodule to be HCC increases with size. The percentages definitely diagnosed as HCC for lesions < 1 cm, 1–2 cm, 2–3 cm, > 3 cm were 68.7%, 91.5%, 94.9% and 97.1% respectively [39]. There is an old saying in liver cancer community that any nodule larger than 1 cm in a cirrhotic liver should be considered as HCC until otherwise proved [40]. From this perspective, we diagnose HCC using US every day in our routine clinical practice. However, we cannot be 100% certain that a nodule depicted by US is indeed HCC, as other contrast-enhanced imaging methods, such as CT or MRI are needed for certification. CT and MRI are used for tumor characterization and staging [14] but can also be used for supplementary nodule detection. Of note, most of the nodules < 10 mm in size detected by US are not malignant [41]. For such tumors, US is valuable in the follow-up strategy, and if a nodule increases in size beyond 10 mm, it should prompt further investigations, such as a CT scan and/or MRI. How to manage these findings is very nicely highlighted in the current European guidelines [14] and it is beyond the purpose of this review. Here, we would like to familiarize the reader with the possible dynamic changes in the US characteristics of such nodules. During the follow-up, the most important US alarm feature is the increase in size. It is not clear how the other above mentioned US features can help in decision making. Whether to continue follow-up until they become greater than 10 mm or to start early additional investigation should be investigated in future studies.

## 4. Performance of US as Screening Tool

A study conducted in the late 80s which aimed to evaluate the usefulness of HCC screening in cirrhotic patients found an overall sensitivity of 78.6%, but only 21.4% for early HCC [42]. Oka H et al. prospectively monitored 140 cirrhotic patients who were screened by US every 3 months and found a sensitivity of 0.68 (95%CI; 0.53–0.82) for early HCC detection. These discrepant results might be explained by a potentially different natural history in Western and Asian patients, the macronodular type of cirrhosis (the main etiology was viral infection), and the use of higher resolution US machines [43]. Later on, in the early 2000s, an increase in the performance of US was noted. Bolondi L et al. reported a prospective study in 313 cirrhotic patients. The sensitivity for overall and early HCC detection was 0.93 (95% CI; 0.84–0.98) and 0.82 (95% CI; 0.70–0.91) [44]. In 2004, Sangiovanni A et al. reported a sensitivity of 0.98 (95% CI; 0.94–1.0) and a specificity of 0.85 (95% CI; 0.81–0.89) for overall HCC detection. Early HCC was found in 54% of the patients with a sensitivity of 0.50; (95% CI; 0.41–0.60) [45].

In a much more recent retrospective cohort study, published in 2018, which evaluated the sensitivity of US compared with cross-sectional imaging in patients with HCC presenting for liver transplantation evaluation demonstrated a pooled sensitivity of 94% (95% CI: 83–98), and a pooled specificity of 94% (95% CI: 89–97). In the early HCC subgroup, the pooled sensitivity was 63% (95% CI: 49–76). However, US sensitivity might have been falsely overestimated by the comparison with CT scans, since US can detect developing tumors at multiple surveillance points [46].

Until now, three meta-analyses were published addressing the role of US as a screening tool (Table 1). The first one [47] assessed the accuracy of different screening tools in HCC using histology as the gold standard. In the subgroup of studies which used the histological findings of the explanted livers as gold standard, the sensitivity and specificity were 48% (95% CI, 34–62%) and 97% (95% CI: 95–98%), respectively. Heterogeneity in this meta-analysis is however an important issue. One study in particular, carried out before 1985 included only symptomatic patients with HCC and HBV prevalence of 36% and 80%, respectively [48]. Two other studies did not report the actual prevalence of cirrhosis among included patients [49,50]. Moreover, two other studies had a prevalence of HCC > 30% [51,52]. And last, most of the studies lacked data concerning the time interval between US and the reference standard.

The second one, published in 2009, found a pooled sensitivity for HCC regardless of staging of 94% (95% CI: 83–98%) and for early HCC of 69% (95% CI 50–83%) [7].

The most recent analysis, by Tzartzeva K et al., found a sensitivity of 84% (76–92%, 95% CI) and 47% (33–61%, 95% CI) for any-stage HCC and early HCC (defined as one tumor < 5 cm or 2–3 nodules, each < 3 cm) respectively [53]. However, two considerations must be made: (1) the inclusion of studies performed in the 1980s and 1990s, when the technology and quality of US were not as developed as today might have decreased the overall sensitivity. Of note, the authors did point out a statistically significant increase in early HCC detection rate from 29.7% in the 1990s to 63% in 2010 (*p* = 0.03); (2) the meta-analysis included studies that compared US with CT/MRI in the same patient. One of the outliers in this meta-analysis [54] compared MRI to US examination. The sensitivity for “early HCC” in this study is reported as 23.3%. This could be explained by the fact that MRI screening was able to detect tumors far earlier in their development than US, thus underpowering the sensitivity of the latter [53].

The data summarized above is the best available to this point yet is far from being perfect. Pooling data only from recent studies (i.e., with enrolment after 2000), ensuring standardized definitions and scenario inclusions, and excluding gross outliers might lead to more reliable figures which could further aide our clinical practice and provide a more realistic overview.

## 5. Factors Affecting US Performance

To this point, the sensitivity of HCC detection is suboptimal and it is the subject of important variation. There are multiple contributors to the accuracy of US screening as illustrated in the subsequent table (Table 2).

**Figure 1:**
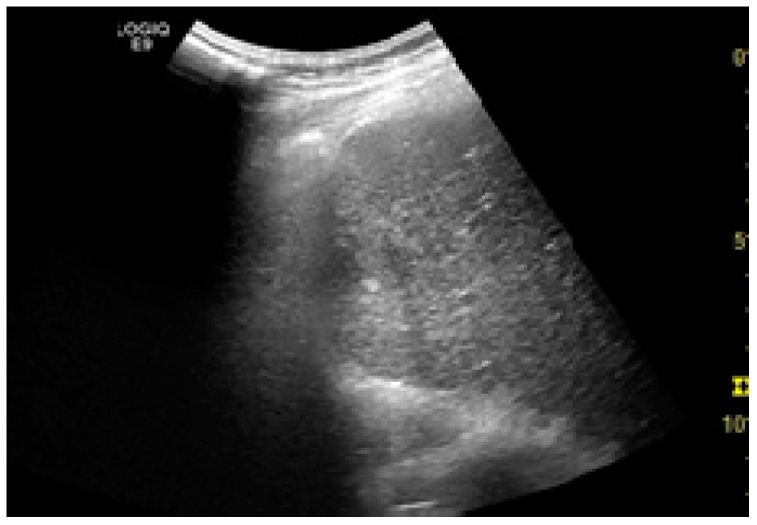
Small hypoechoic hepatocellular carcinoma (HCC) in a cirrhotic liver. US evaluation.

**Figure 2:**
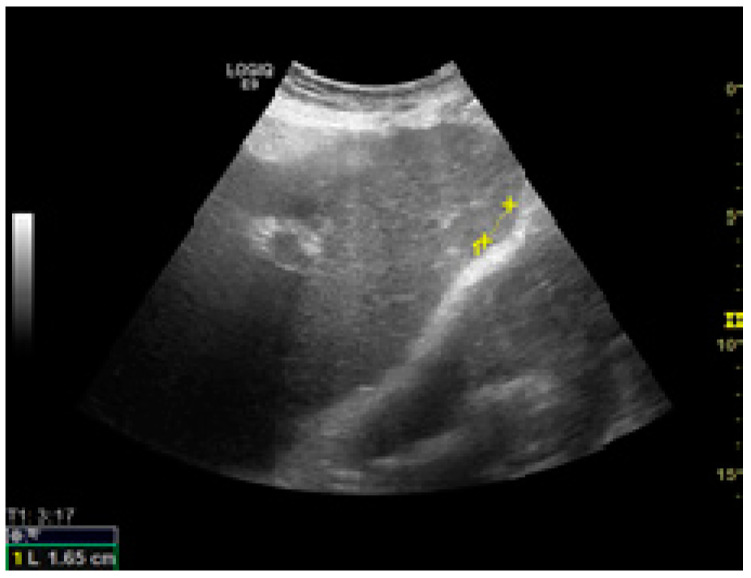
Small isoechoic HCC, with a subcapsular location. US evaluation.

Among the factors associated with US detection performance, tumor size is one of the most important predictors, as larger nodules consistently lead to higher sensitivities. In one recent study, the sensitivity of US was 90%, 84%, 76%, and 65% for nodules larger than 4 cm, 3–4 cm, 2–3 cm, and 1–2 cm, respectively [46]. Infiltrative tumors are much more difficult to detect via US and are more often associated with surveillance failure [31,55]. In one study on 304 HCC patients who received regular surveillance with US and AFP, the failure rate was significantly higher for patients with infiltrative type tumors (57.1%) compared to nodular tumors (2.1%) [55]. Regarding echogenicity, isoechoic and faint hypo/hyperechoic lesions may escape a regular US evaluation (Figure 1 and Figure 2) [31,37]. The US visualization of nodules located in a deep or subcapsular position and/or near lung tissue (segments VII, VIII, and IVa) might be very difficult, or sometimes even impossible [31,56].

The underlying liver disease has a strong influence on the accuracy of US surveillance. The detection of early-stage tumors may be challenging when numerous regenerative or dysplastic nodules are present in the liver [56]. In one study, parenchymal macronodularity was a significant predictive factor for surveillance failure, especially in patients with HBV infection [55]. However, a more recent report suggested a lack of relationship. The discrepancies might be explained by the differences in design and follow-up, as patients with parenchymal macronodularity frequently underwent CT or MRI to detect liver cancer despite negative results on surveillance US [56].

NASH-related liver fibrosis/cirrhosis substantially decreases the sensitivity of surveillance US (59%), in comparison to other etiologies (84%) [34,46]. Even if the addition of AFP (cut-off level > 20 ng/mL) increases the US sensitivity to 72%, the figures remain lower in comparison to other etiologies (91%) [46]. Regarding the severity of liver disease, there are no robust available data to support a lower accuracy of US surveillance in a more advanced liver disease [46]. However, there are reports suggesting that the severity of cirrhosis assessed either by Child–Pugh class or MELD score might be an important predictor of ultrasound quality [31,57].

US quality is also influenced by patient-related characteristics, which can be modifiable or not. Among them, the most important contributor to HCC detection failure appears to be obesity. Its impact on the quality of screening US exams is multifaceted. On one hand, obese patients tend to have NASH, either as a primary or as an associated liver disease etiology, which, as discussed above, decreases accuracy due to liver-related factors. On the other hand, an increased body mass index (BMI) hinders a proper examination, regardless of liver disease etiology, leading to a 10% decrease in sensitivity even in non-NASH patients (77% for BMI ≥ 30 kg/m^2^ vs. 87% for lean patients) [46]. Furthermore, BMI appears to be inversely correlated with the quality of the examination, as less than half of class 2 and morbidly obese patients benefited from an adequate report, according to one study [57]. This drop in sensitivity might be explained by abdominal adipose tissue disposition, abnormal respiratory amplitude, and overall thick abdominal wall. Among the modifiable factors, intraluminal gas is a well-recognized hindrance to acquiring an adequate echogenic window; leading to HCC surveillance failure in up to 50% of cases in which adequacy was not attained [56]. This aspect is of particular interest as a large proportion of the screening examinations are performed in an outpatient setting and standardized conditions might be difficult to reach. Thus, inadequacy should prompt a re-visit. Another patient-related variable which might decrease sensitivity could be an altered liver anatomy, either by prior non-HCC liver surgery, trauma, or interventional procedures. Yet, evidence is only empirical to this point and the decrease in sensitivity was marginal and non-significant in available reports [46].

While the three aforementioned factors are related to the screening recipient, the following contributors depend on the screening provider, expanding in scale from the operator, up to the medical system and regional surveillance protocol. Available data trivially suggests that any US operator is better than no operator. Same-operator or same-center consistency does not appear to significantly influence surveillance failure rates [58]. This is further reinforced by the results from the ITA.LI.CA database, which amassed up to 1170 patients in the span of two decades [31]. According to the Italian experience, the type of center (primary vs. tertiary) did not affect surveillance failure. Moreover, the same dataset did not find a significant difference in detection rates according to the period in which the screening was performed (late 1980s and 1990s vs. early 2000s). This suggests that, despite notable technical advancements (US machines, probes, modules, and applications), detection rate did not improve. Yet, this statement is contradicted by Khalili K et al., which, based on a more recent experience reported that detection rate did significantly increase in the latter part of the 2000s decade [59]. However, these relatively optimistic findings might be counterbalanced by a recently published Korean report, which revealed substantial discrepancies in screening quality between healthcare providers. Furthermore, the level of physician knowledge and education was concerning, with a substantial number of US operators lacking essential training [60].

Concerning the detection rate of early HCC, US surveillance at least every 6 months led to a sensitivity of 70.1% (95% CI: 55.6–84.6) which was significantly better than the sensitivity of 50.1% (95% CI: 40.0–59.2) in the studies performing surveillance on an annual basis (*p* = 0.001) [7].

## 6. How to Improve US Screening?

### 6.1. An Adequate Ultrasound Examination and Potential Targets for Improvement

The features which make US the best available surveillance tool can also transform it in a double-edged sword. Thus, each of its strengths might also become a potential drawback if it is not properly accounted for. The complexity of the technique might lead to errors and inadequacy at multiple levels, starting from how a visit is scheduled, to US-machine technical aspects, operator-related issues, per se scanning quality, and, not least, patient characteristics. Therefore, to fully reap the benefits of an US surveillance examination, it is mandatory to ensure that an adequate visualization was obtained.

**Figure 3:**
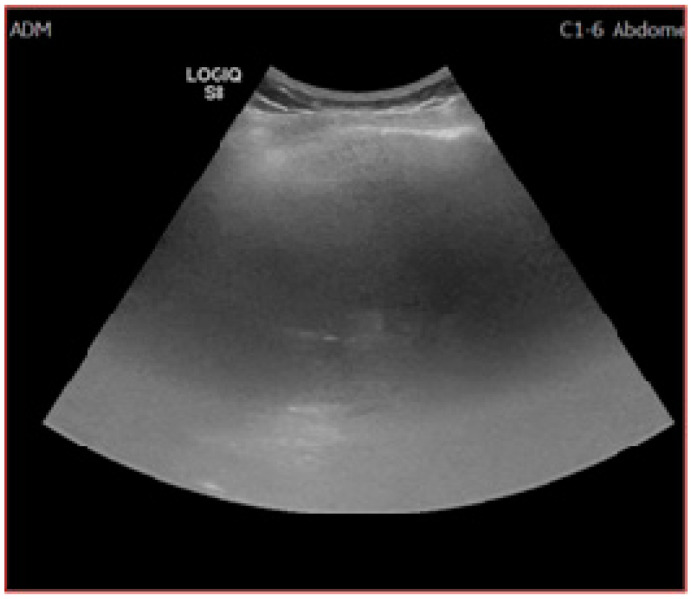
Inadequate US examination. Posterior and superior part of the right lobe could not be visualized due to extensive steatosis and rib shadowing.

As reported by a recent quality assessment study [57] only 66.5% of the reviewed US exams were deemed as “definitely adequate”, which obviously leaves significant room for improvement. Unfortunately, to this point there are no consensus-accepted benchmarks for an adequate examination, which shifts this discussion from the field of “evidence-based” to the realm of practice-derived epistemological subjectivism. According to the study, the most common obstacles towards a proper US report were insufficient parenchymal visualization (less than two-thirds) due to poor beam penetration and excessive rib shadowing (Figure 3). Liver heterogeneity and bowel gas affected the examinations to a lesser extent. The most significant predictors for poor quality were in-patient status, male gender, NASH and obesity, alcohol-induced liver disease, and Child–Pugh B and C [57].

A large-scale Korean quality assessment report [60] revealed some glaring aspects with regards to adequacy. The overall suboptimal level at which ultrasound was performed was determined by numerous factors, the most strikingly deficient being physician education. To increase the exam quality, the Korean Radiology Society, along with the National Cancer Center developed guidelines for quality management and a quality assurance questionnaire. There was a wide array of items investigated, ranging from equipment and personnel, to education, report form and actual image analysis—proper device setting, artifacts, and standardized images. Of particular clinical relevance might be the acquisition of ten standardized images, as summarized in Table 3.

Improving the overall quality of US HCC surveillance is a daunting task and should probably lead to a concerting effort to reach an adequate standard for each operator and exam. One key component might be specific operator certification and image standardization. A second important component could be recognizing that even for best possible operator, not all images are equal. Thus, a report should at least contain the operator assessment of the exam, as multiple factors can affect its value. A low-rated exam should consequently prompt either a revisit or a complementary examination method, on a case-by-case assessment.

Not least, in the current era of rapid narrow artificial intelligence development, a potential solution might come from deep-learning algorithms and radiomics to further aid clinicians in assessing image adequacy and increase nodule detection [61,62].

### 6.2. Defining Classes of Risk and Developing Imaging Strategies According to the Risk

HCC screening is a lifesaving intervention, increasing the chance of being alive after five years of follow-up by 37% [9]. However, the efficiency of the screening remains poor, provided that the real-life adherence to the program is only 40%, as revealed by a large meta-analysis [63].

As a matter of fact, each level of the screening program could and should be improved to increase its efficiency (Table 4).

One of the trending solutions for improvement is to refine and personalize the risk for developing HCC. Thus, several clinical scores have been developed and validated (Table 5).

The GALAD score, seems to be superior to US for HCC. The GALAD score discriminated patients with HCC from patients with chronic liver diseases with an AUROC greater than 0.90 [67]. Moreover, GALAD discriminated patients with HCC from those with other primary liver cancers, patients with treated chronic viral hepatitis, or patients with NASH regardless of liver fibrosis.

The combination of GALAD and US (GALADUS score) further improved performance, achieving an AUROC of 0.98 (95% CI, 0.96–0.99; cutoff −0.18, sensitivity 95%, specificity 91%) [69].

Very recently, the aMAP risk score (ranged from 0 to 100) was identified and validated in a large cohort of patients with chronic hepatitis, regardless of etiology and ethnicity. The aMAP score can stratify patients in three risk groups (Low < 50, medium 50–60, and high > 60). Patients with aMAP < 50 had an HCC incidence of <0.2% per year, meanwhile in the high-risk group (aMAP > 60) the incidence of HCC was 1.6–4.0%/year [68].

Nowadays, in the era of personalized precision medicine, all the pieces of the puzzle can be refined, reshaped, and rethought for better care and improved outcomes.

The most important thing we, as caregivers, must not forget is that, for the target population, HCC is one of the many competing risks for poor outcome and mortality [70,71,72]. In this respect, the Baveno VI consensus introduces the concept of compensated advanced chronic liver disease (cACLD), which is an important one, because it defines the moment in the natural history of chronic liver disease from where the risk of decompensation and/or HCC significantly increases [70]. The diagnosis of cACLD relies on liver elastometry (i.e., liver stiffness measurement—LSM, by vibration controlled transient elastography): a LSM < 10 kPa rules out cACLD, while values > 15 kPa are highly suggestive of cACLD [73]. More than that, a LSM > 21 kPa is highly suggestive for clinically significant portal hypertension [74] and bears a similar two-years predictive power as a hepatic venous pressure gradient (HVPG) > 10 mmHg for portal hypertension-related mortality and all-cause liver mortality (including HCC) [75].

In terms of predictive accuracy, LSM was demonstrated to be associated with an increased risk of developing HCC during a three-year follow-up period in both HBV [76] and HCV [77] infected patients. Patients with a baseline LSM > 8 kPa (for HBV) or >10 kPa (for HCV) had a higher incidence of HCC, and the relative risk was higher as greater baseline LSM values were recorded (Table 6).

In alcoholic liver disease (ALD), the association between a higher LSM and HCC development was also observed. The cumulative five-year incidence rate of HCC was 26.3 in patients with compensated ALD and baseline LSM > 11.5 kPa (as compared with 0.4 in those below this arbitrary threshold) [78]. The incidence of HCC in NAFLD/NASH is lower than in HCV (2.4 vs. 4% during 38 months of follow-up) [24]. Although HCC can occur in the absence of cirrhosis in up to 34.6% of cases [79], the incidence is 25-fold higher in patients with advanced fibrosis [24]. Although LSM is not reliable in up to 20% of patients with NAFLD/NASH, it can accurately detect advanced fibrosis (F3) with 85% accuracy (>90% sensitivity and > 90% specificity) for values >10 kPa [80].

US is a continually evolving and growing field. Better devices with multiple capabilities and examination modes are becoming widely available. In this context, the availability of contrast-enhanced ultrasound (CEUS) is expected to increase. There are two types of contrast agents available, both being a suspension of inert gas. One is Sulphur hexafluoride (SonoVue^®^, Bracco, Milan, Italy)—a purely intravascular agent, and the second is Perfluorobutane (Sonazoid^®^, GE Healthcare, Norway)—which allows a stable and long-lasting Kupffer phase in addition to the vascular phase imaging. SonoVue is used globally, having both FDA and EMEA clearance, while Sonazoid is approved only in China, Japan, Korea, Singapore, Taiwan, and Norway.

CEUS is not currently accepted as a diagnostic tool for HCC [13,14], because of differential diagnosis issues with intrahepatic cholangio-cellular carcinoma—ICC [81]. However, the efforts to unify and systemize the CEUS reporting in cirrhotic patients with focal liver lesions carried on by the American College of Radiology—the LIRADS system [82] have clarified many of the issues. Apart from LIRADS 4 and 5—which are highly suggestive and typical for HCC, a new LIRADS M (malignancy) class is identified. According to these criteria, a liver lesion which is hypo enhanced in the venous and late phase (usually earlier than 60 s) after SonoVue injection has a high probability of being malignant. In fact, it seems that CEUS is better than CT to identify M lesions, most likely because the target nodule was already depicted on grayscale US [83]. Although the characteristics of arterial phase can differentiate between HCC and ICC [84], both malignancies are hypo-enhanced in the venous and late phase.

Similarly, the parenchymal (Kupffer) phase (10–60 min after Sonazoid injection) shows marked hypo-enhancement in HCC. This particularity makes Sonazoid CEUS a suitable method for HCC screening. Indeed, Sonazoid CEUS screening raised the HCC suspicion earlier (3.5 vs. 4.4 years) and detected smaller (13 vs. 16.9 mm) nodules, as compared with B-mode US screening. It also showed excellent sensitivity and negative predictive value (100%) and very good specificity (96.1%) [85]. However, another study with a different design did not find a better HCC detection rate in Sonazoid CEUS screening group as compared with conventional US (2/524 more patients detected). Still, it significantly improved the false referral rate (17/524 fewer patients erroneously referred) [86].

Altogether, it looks that adding CEUS to conventional B mode US—especially the late phase with either contrast agent type, might increase the accuracy of HCC screening, but further prospective randomized trials are needed to demonstrate this.

**Figure 4:**
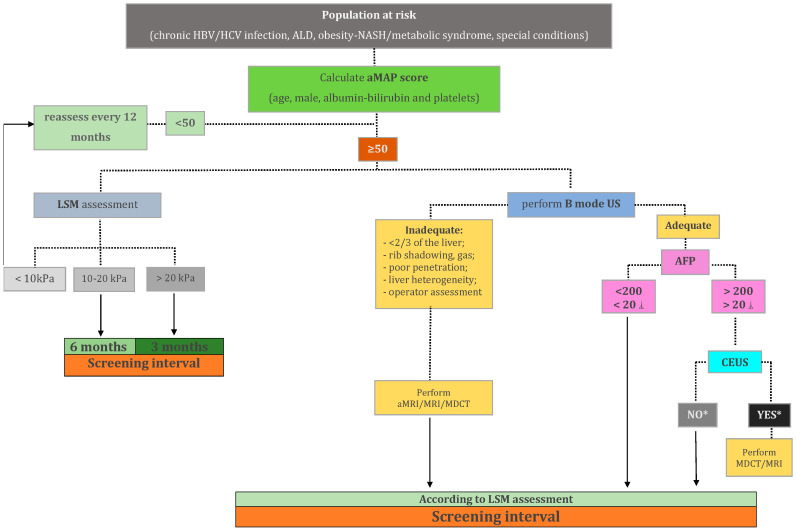
Proposal for a multiparametric personalized hepatocellular carcinoma screening in patients with cACLD. HBV—hepatitis B virus; HCV—hepatitis C virus; ALD—alcoholic liver disease; aMAP—hepatocellular carcinoma risk score, as described by Fan R et al. [68]; LSM—liver stiffness measurement; US—ultrasonography; aMRI—abbreviated magnetic resonance imaging; MDCT—multidetector computed tomography; AFP—alpha-fetoprotein; CEUS—contrast-enhanced ultrasonography; ⸸ 20 ng/mL cut-off value to be used for patients with a prior history or ongoing treatment for chronic viral hepatitis or curative-intent treatment for hepatocellular carcinoma; * a liver lesion which is hypo enhanced in the venous and late phase.

HCC screening and surveillance in patients with cACLD is far from being a closed subject. We should adapt our strategies as new data became available, to increase the efficacy of the program without raising the subsequent costs. One possible approach, which includes a multiparametric assessment, is depicted in the figure below (Figure 4).

Of course, the algorithm above should come with an explanatory note. It is nowhere near a perfect solution, being merely a proposal generated by mixing the aforementioned raw data with empirical rationale. To this point, no firm, hard-data-backed recommendations can be made beyond a negative B mode US examination. Therefore, the clinician is left facing a critical choice between overconfidence (in US) and overscreening. The challenge of overconfidence can be addressed by promptly recognizing a “prone-to-failure” B mode examination, which typically involves low-quality imaging in a high-risk patient. As previously discussed in Section 6.1, there are no clear-cut benchmarks for a high-quality exam. However, being unable to adequately visualize at least two-thirds of the liver parenchyma due to insufficient beam penetration, shadowing, or gas, along with a subjective assessment should raise important red flags. A typical high-risk patient is, as discussed in Section 5, either obese with or without NASH fibrosis and cirrhosis or having a macronodular, profoundly heterogeneous liver, as frequently encountered in HBV cirrhosis. On the other hand, there are also challenges with regards to overscreening. Even after a poor US scan, the clinician is still facing a negative result, which places the entire scenario in the realm of screening, rather than in the realm of diagnosis. However subtle, this distinction is important, as the pre-test detection probability differs significantly between the two settings. This is the main argument for including aMRI as a possible tool in the screening scenario, while potentially reserving MRI and MDCT for diagnostic purposes. However, a valid point can be made for a “baseline” hepatocyte-specific contrast-enhanced MRI following a negative, but low-quality B mode exam. This approach might effectively rule out any existing nodules, yielding both high sensitivity and specificity regardless of patient condition, thus providing a “2 in 1” screening and diagnosis solution. However, it comes at a higher cost, a theoretically higher risk, and a longer examination, all of which represent significant caveats in a screening setting. The particularities and performance of specific MRI protocols are briefly discussed in the upcoming Section 6.3. To this point, the discussion regarding the best algorithm is wide open, as new data can significantly tilt the approach in one way or the other.

### 6.3. Sectional Imaging as an Alternative to Ultrasound in the Screening for HCC

US is the recommended surveillance tool for patients with liver cirrhosis at risk for developing HCC. Several limits of US have been discussed in the previous sections. Different from US, sectional imaging techniques are less operator-dependent and less influenced by the patient status [87]. Computed tomography was proposed as a screening tool with promising results [87]. However, the findings of this study are influenced by the small number of patients enrolled. Irradiation and usage of contrast media make computed tomography less desirable as a screening tool. Unenhanced CT is of no use, neither in HCC detection, nor in characterization of nodules in the cirrhotic liver. Another study reports excellent specificity of CT, but with a low sensitivity [88]. Mediocre sensitivity is a major drawback for a screening technique. Later on, several studies confirm the efficacy of MRI as a surveillance tool for HCC [54,89]. The main disadvantages of MRI are the limited number of machines, the long duration of the examinations, high costs, and the usage of intravenous contrast media. The study by Kim et al. reported very good sensitivity and significantly fewer false-positive results of contrast-enhanced MRI as compared to US. In this study, biannual MR with hepatocyte-specific contrast media was used, which significantly increases the screening costs. Another paper, by Yu et al. reports rather weak sensitivities of both CT and MRI in the detection of HCC nodules [90]. This paper compares HCC detection with the detection of nodules found on the explanted liver. Whereas nodule-based sensitivity is mediocre for both CT and MRI, patient-based sensitivity is very good for both techniques. Recently, abbreviated MRI (aMRI) protocols for the detection of HCC have been proposed. In some of them, injection of contrast media is also recommended. Park et al. have proposed an aMRI protocol with injection of liver-specific contrast media and image acquisition only in the late, hepatobiliary phase. Such protocols reduce the time of acquisition, however not significantly reducing the costs at the same time [91]. Another paper concluded that abbreviated, contrast-enhanced MRI had equivalent results with a complete, contrast-enhanced MRI for the detection of HCC nodules in cirrhotic patients [92].

**Figure 5:**
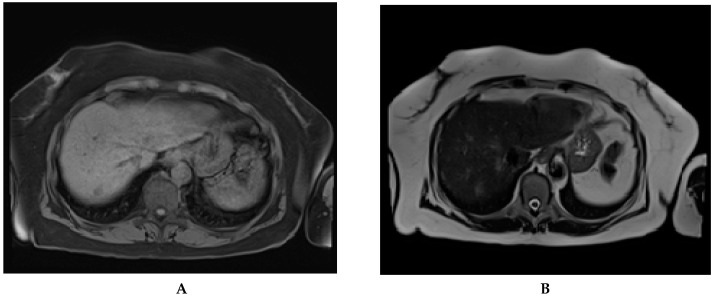
Abbreviated magnetic resonance imaging done for HCC surveillance in a 74 years old cirrhotic patient. A newly discovered nodule, in segment 7, is to be seen on the T1 vibe fs tra sequence (**A**) and T2 haste tra sequence (**B**). The nodule was later confirmed and classified as LI-RADS 5 by CEUS.

Some papers proved that non-contrast MRI was also sensitive in the detection of HCC nodules [47,90]. Performing a non-contrast MRI will reduce both examination time and costs [54]. Recent non-contrast-enhanced, abbreviated MRI protocols showed comparable results with contrast-enhanced CT and MRI in liver nodule detection (Figure 5) [54].

Recent studies report a sensitivity of aMRI in the detection of HCC nodules which is comparable to that of liver-specific enhanced MRI or ultrasound performed by experts [93]. Interestingly, non-contrast MRI proved to have higher specificity and lower false positivity rate as compared to contrast-enhanced MRI for the detection of small sized HCC. This may be due to the presence of foci of transient arterial hyperenhancement, which may be misdiagnosed as HCC, and which are visible only after contrast media injection. Sensitivity and specificity of sectional imaging techniques in detection of HCC nodules are listed in Table 7. There are several on-going clinical trials which compare the detection rate of early and very early HCC by ultrasound and abbreviated, non-contrast MRI [54,94].

To conclude, both CT and MRI can be used as an alternative to US for HCC detection in the cirrhotic patient. High costs and toxicity for the patient due to the usage of ionizing radiations and contrast media injection are limitations of these techniques. To reduce costs and time of examination, several aMRI protocols have been proposed. Non-contrast aMRI showed comparable sensitivity with contrast-enhanced MRI for the screening of HCC nodules on the cirrhotic liver. The lower costs as compared to contrast-enhanced MRI and the reduced time of examination of non-contrast aMRI make this technique suitable to be used as complementary to US HCC screening. As mentioned before, there are some categories of patients (e.g., obese patients and/or NASH patients) in whom US screening is inappropriate. We believe, that for these patients the future has come. We should therefore abandon US as a screening tool and try to use more often the novel sectional imaging techniques.

## 7. Conclusions

We would like to conclude with some answers to questions that will possibly emerge for our future readers of this manuscript. US or sectional imaging in HCC screening? Definitely US, well, not for all but for the large majority. Moving forward or falling forward? There are several methods that can improve US screening (i.e., increase patient access to screening, familiarize the patients with the diagnosis of cirrhosis and its related complications, educational programs for physicians who perform US screening for HCC in cirrhosis, labeling an US examination as adequate or inadequate, etc.)—fall forward! Whenever US is deemed to be inadequate (either because factors related to screening recipient or screening provider) sectional imaging is the way to go—move forward!

What is the best screening interval: move forward or fall forward? US every 6 months as a screening tool in all our patients—fall forward! A personalized approach? One example could be the multiparametric personalized HCC screening model proposed in our review (where AFP, LSM, and risk scores for HCC are important in clinical decision making)—move forward!

Ultrasound might not be a perfect screening tool, but for the time being is the best available, so we must improve it and fall forward. Nevertheless, we should also move forward when possible and adapt our strategies as new data became available.

## Figures and Tables

**Table 1:** Meta-analyses addressing ultrasound-based HCC screening.

				Any Stage HCC		Early HCC	
Author and Year	Period	Studies Included	Patients Included	Se %, (95 CI)	Sp % (95 CI)	Se% (95 CI)	Sp% (95 CI)
Colli et al. 2006 [47]	1984–2003	14	7347	60.5 (44–76)	96.6 (95–98)	NA	NA
Singal et al. 2009 [7]	1985–2003	6 * 13 **	2984 * 3567 **	95 (89–98)	91 (77–97)	69 (50–83)	NA
Tzartzeva et al. 2018 [53]	1990–2016	31 * 15 **	12997	84 (76–92)	NA	47 (33–61)	NA

HCC–hepatocellular carcinoma; * Studies that assessed the development of overall HCC; ** studies that assessed the development of early HCC; Se—sensitivity, Sp—specificity, NA—not available/applicable.

**Table 2:** Factors affecting the efficacy of US surveillance.

1. Lesion (size, margins, echogenicity, location)
2. Underlying liver disease (the aspect of parenchyma, etiology, severity)
3. Patient status (bodyweight, abdominal fat, intra-abdominal gas, previous surgery)
4. US expertise
5. Quality of US machines
6. Modality of screening (visit frequency)

**Table 3:** Standardized ultrasound (US) images required for an adequate examination, adapted after Choi et al. [60].

Key Morphological Feature	Standard Image
Vasculature	Transverse plane of right and left portal veins
	2. Subcostal scan of hepatic veins at hepatic dome
Biliary structures	3. Longitudinal scan of extrahepatic duct
	4. Longitudinal scan of gallbladder
Left liver lobe	5. Longitudinal scan
	6. Transverse scan
Right liver lobe	7. Transverse scan
	8. Intercostal scan—including hepatic vein
	9. Intercostal scan—including portal vein
	10. Right hepatic dome

**Table 4:** Current recommendations and possible solutions for improvement of the HCC screening strategy.

Elements of the Screening Program	Current Recommendations	Possible Ways for Improvement [Sherman 2019]
Population at risk	Patients with advanced fibrosis or cirrhosis (Child A and B), regardless of etiology; Child C cirrhotics awaiting liver transplantation; HBV+ patients with intermediate /high PAGE-B risk scores [14]	Refine the risk assessment (when should screening start); Refine/individualize risk scores according to clinical scenarios (consider the effect of etiology/geography); Develop reliable (universal) biomarkers;
Screening tests	B mode ultrasound (US); [14,15,16,24] AFP (≥200 ng/dl) [16]; DCP, AFP-L3 [15]	Make use of technical advances in US assessment and emerging US-based examination modalities (elastography and contrast-enhanced US—CEUS); Determine the optimal level of the screening tests’ sensitivity that would impact cure rates and survival;
Screening interval	6 months [14,15,16,24] 3–4 months (extremely high-risk patients) [15]	Individualize according to risk and clinical scenario;
Recall procedures		Improvement and standardization of confirmatory tests (cross-sectional imaging and/or biopsy)

**Table 5:** Clinical risks scores validated for HCC development.

Score	Author, Year	Clinical and Laboratory Parameters	Other Parameters
REACH-B	Cheng et al., 2006 [64]	age, gender, serum levels of ALT, HBe antigen status, and HBV DNA level	
CU-HCC	Wong et al., 2014 [65]	age, albumin, bilirubin, HBV DNA	Liver Stiffness Measurement (Fibroscan)
PAGE-B	Papatheodoridis et al., 2016 [66]	age, gender, platelets	
GALAD	Berhane et al., 2016 [67]	age, gender, AFP-L3, AFP, and DCP	
aMAP	Fan et al., 2020 [68]	age, gender, albumin, bilirubin, platelets	

ALT—alanine aminotransferase; HBe—hepatitis B e antigen; HBV—hepatitis B virus; FP-L3—Lens Culinaris agglutinin-reactive Fraction of Alpha-Fetoprotein; DCP—des-gamma-carboxyprothrombin.

**Table 6:** Increased risk of developing HCC during three years of follow-up, according to baseline Liver Stiffness Measurement.

Etiology	Liver Stiffness Measurement Intervals (kPa)	Hazard Risk for Developing Hepatocellular Carcinoma
Hepatitis B	8	3.0
	13	4.6
	18	5.5
	23	6.6
Hepatitis C	10	16.7
	15	20.9
	20	25.6
	25	45.5

**Table 7:** Sensitivity and specificity of sectional imaging techniques in the surveillance of HCC.

Study	Year	Technique	Sensitivity	Specificity
Kim, et al. [95]	2014	Non-contrast MRI	91%	77%
Pocha, et al. [87]	2013	Contrast-enhanced CT	87%	87%
Van Thiel, et al. [88]	2004	Contrast-enhanced CT	70%	100%
Kim, et al. [54]	2017	Liver-specific contrast-enhanced MRI	83%	Not available
Yu, et al. [90]	2011	Contrast-enhanced CT	65%	96%
Yu, et al. [90]	2011	Contrast-enhanced MRI	72%	87%
Chan, et al. [93]	2019	Non-contrast, abbreviated MRI	85%	93%
Besa, et al. [96]	2017	Contrast-enhanced abbreviated MRI	80%	87%
